# Course‐based undergraduate research experiences in a remote setting: Two case studies documenting implementation and student perceptions

**DOI:** 10.1002/ece3.6916

**Published:** 2020-10-30

**Authors:** Samuel B. Fey, Meredith E. Theus, Aaron R. Ramirez

**Affiliations:** ^1^ Department of Biology Reed College Portland OR USA

**Keywords:** independent student research, inquiry‐based pedagogy, remote learning

## Abstract

Inquiry‐based components of ecology curricula can be valuable, exposing students to what it means to *do* science, from conceiving of a meaningful question to effectively disseminating results to an audience. Here, we describe two approaches for implementing independent, remote research for undergraduates enacted in the spring semester of 2020 at Reed College in Portland, OR, reporting case studies from an intermediate‐level ecology course and an interdisciplinary environmental science course. We report on both the challenges as well as the novel opportunities for independent research projects in such a setting, the details of how projects were implemented, the tools and resources that may help facilitate such endeavors, as well as perceptions on the effectiveness of this endeavor by students. As institutes of higher education continue to operate in an online learning environment, we hope these materials help spark a discussion about how to engage in meaningful research experiences as part of coursework in the COVID‐19 era and beyond.

## Graphical abstract

We report on both the challenges as well as the novel opportunities for independent ecology research projects in a remote learning setting, the details of how projects were implemented, the tools and resources that may help facilitate such endeavors, as well as perceptions on the effectiveness of this endeavor by students.

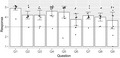

## 1 INTRODUCTION

Similar to many institutions, Reed College (Portland, OR) transitioned to a remote leaning environment in Spring 2020, with 5 weeks remaining in the semester. Historically, many of Reed's upper division courses in math and natural sciences—including Ecology (Biology 301) and an interdisciplinary Environmental Science (ES 300) Junior Seminar (both full credit, one semester courses)—culminate in students pursuing independent research projects (IPs), a type of course‐based undergraduate research experience, CURE (Linn et al., 2015). In conducting IPs, students conceive of an original research question, design and implement an appropriate research plan, work individually or in small teams, and conclude by communicating their findings to an audience of their peers and faculty member. A remote version of these IPs was enacted in Spring 2020 in response to the novel academic setting initiated by the COVID‐19 pandemic.

Pursuing IPs amidst the challenges of living through the COVID‐19 pandemic are apparent. At the onset of this endeavor, we identified the following (explicitly not exhaustive) primary challenges associated with students living and studying remotely: (1) no access existed to our typical teaching laboratories, limiting instrumentation and software access; (2) limited access to known field sites in the areas surrounding students—both due to geographic distance between students and our campus, geographic variation in the extent to which “stay at home” mandates exist, park closings, and additional common sense safety measures. Lastly, and not unique to this activity, (3) students experience great variation in housing stability, Internet access, and added responsibilities that conflict with time to devote to academic pursuits.

However, these challenges were accompanied by several serendipitous opportunities inherent to the fields of ecology and environmental science in the 21st century. First, producing high quality science with a small budget and only low‐tech equipment is feasible within ecology. Undergraduate students are often amazed at the ingenuity of using household items, such as leaf blowers (Donihue et al., 2018), to complete research projects. Second, ecological research can sometimes be accommodated in an abbreviated period of time—for example, using space for time substitution (Blois et al., 2013). Thirdly, many ecology/environmental science projects in the 21st century are conducted in remotely distributed research teams (Hampton & Parker, 2011). Finally, ecology, and teaching in ecology, relies heavily on multiple lines of inference to understand how ecosystems function, including experiments, models, and observations (Cottingham et al., 2017). The combination of pre‐existing ecology data and access to multiple approaches to leverage and engage with this data (e.g., meta‐analysis, parameterizing quantitative models with existing data, formulating new quantitative models) allows for some research to be completed without ever having to venture outside (Auker & Barthelmess, 2020).

Below we report this experience as a teaching case study of two recent “cases” or courses at the primarily undergraduate institution Reed College. In both cases, faculty provided a 5‐week schedule for the independent projects, which included three phases of: planning, implementation, and dissemination of findings (Table 1). Below we elaborate on the timeline and activities associated with each phase and provide a summary of student evaluations of the experience. We conclude by highlighting the feasibility of such projects and identify the remote learning tools that can be used for pursuing future independent research projects in remote learning environments.

**TABLE 1:** Stage goals, activities, and focal skills associated with each phase of projects

Phase	Activity	Focal science skill
Project Planning	Individual brainstorm Mini proposal presentations Final proposal presentations and finalizing groups	Reading primary literature Making natural history observations Critical discourse Connecting observations with questions and hypotheses Experimental design Science communication
Project implementing	Independent time to work on problems	Implementing science Teamwork
	Zoom check‐ins with faculty	Statistical inference Graphical inference Data interpretation
Project dissemination	Online project symposium	Science communication Collaborative writing Critical discourse Scientific collegiality

## 2 CASE STUDY 1: ECOLOGY (BIOLOGY 301)

### 2.1 Overview for Bio 301 CUREs

Bio *301* is a full semester course consisting of two 80‐min lectures and one 4‐hr laboratory each week. Its prerequisites are two semesters of lecture/laboratory introductory Biology. This course was attended by 12 students: 6 students with junior standing, 5 students with sophomore standing, and 1 student with senior standing. Declared or anticipated majors included Biology (*N* = 6), Environmental Studies (*N* = 3), Russian (*N* = 1), English (*N* = 1), and Undecided (*N* = 1). Two students were unable to participate in any aspect of the CUREs, leaving 10 participating students separated into two laboratory sections. All course activities address the primary learning goal of offering rigorous exposure to the major theories and concepts that define the field of ecology and actively engage students in the process by which theories are tested, falsified, and refined. We report below how we used 5 weeks of laboratory to conduct independent projects based on three phases (Table 1).

### 2.2 Project planning modifications

The central goal for weeks 1 and 2 was to develop a venue for students to conceive of a feasible independent project, assemble into a group (if necessary), and transition from an idea to an actionable plan. This process was primarily conducted using video conferencing software (Zoom Communications Inc.), in conjunction with the instructor emailing students to share similar project ideas between laboratory sections (see above) to foster group formation. During week 1 over Zoom, Students reported on a single feasible project idea and delivered a short (~5–7 min) prepared, albeit informal presentation for pitching these proposals to their classmates and the instructor. Students reported (1) what is their motivation for pursuing this project, (2) what would the data (or data product) collection entail, (3) what are the associated hypothesis(es)/prediction(s), or expectation(s)? and (4) how they might statistically analyze collected data. Previously in the semester, students shared strategies for choosing a project, such as extending what was completed in laboratory from a previous week or testing a concept or theory covered in the lecture component of this course (see Appendix 1 for additional project details), that helped “prime the pump” (D'Avanzo, 2003) for more engaged participation. Following presentations, we held brief (~10 min) laboratory‐wide discussions where students asked their peers clarifying questions and provided suggestions for improving the proposed projects in a videoconference format, allowing the often neglected component of critical discourse (Osborne, 2010). After discussions, students were given additional time to remotely discuss the feasibility of working together (group fusion was explicitly encouraged), and were instructed to spend the time during the ensuing week collecting pilot data, reading the literature, and honing in on a specific design for implementing these projects, including identifying all materials needed to conduct the research.

During week 2, individuals or groups again prepared and delivered short (~5–7 min) Zoom presentations describing how the previous week's activities had shaped the project development which was followed by a ~10‐ to 15‐min discussion of the project by all participants of the laboratory using videoconferencing. Group fusion was again encouraged during this time. All groups were required to submit a formal plan by the end of the week that included the research question, the proposed plan to address this question, a plan for analyzing results, and a complete materials list. The goal by the end of week 2 was to have all students assembled into groups with a feasible path for completing their projects (Appendix 2). The format of weeks 1–2 were largely unchanged from an in‐person offering of this course, but included the additional modifications of spending time explicitly considering the feasibility of conducting the project due to safety considerations, geographic constraints, and potential logistic and instrumentation constraints. Importantly, despite emphasizing the opportunities that exist for questions to be addressed as a result of students living in different geographic locations, only one group chose to work in pairs (Appendix 2), highlighting the challenges of coordinating team efforts when participants are dispersed across locations.

### 2.3 Project implementation modifications

Weeks 3 and 4 were exclusively dedicated to implementing projects. Despite the instructor highlighting the use of existing and “open” ecological data, all projects used empirical tools to collect new data, indicating a preference for “hands‐on” research experiences, even with material and travel limitations. Because several projects required equipment to complete planned project activities, we quickly shipped materials from campus starting in weeks 1 and 2, or ordered materials online to be delivered directly to students (Appendix 2). In this remote setting, we observed that time commitments outside of class time make extra group meetings challenging; therefore, we dedicated scheduled laboratory time to allow for students to meet and work on IPs. To help maximize mentoring opportunities during this phase of the project, regular (e.g., during laboratory) and irregular check‐ins outside of class time and office hours were made available. These more frequent meetings proved helpful to field questions that arose during data collection and analysis and helped students find the right scope of the project given existing constraints. Five out of 10 students embarked on a project working with a study system that they had previously worked with in laboratory, suggesting that the previous in‐person laboratory instruction was not required for implementing projects. Five out of 10 students used experimental approaches to answer an ecological question, and 5 used observational approaches. Depending on additional safety concerns in subsequent years, imposing requirements to use one specific approach would constrain project choices (Box 1).

Box 1 **Weighing the merits and drawbacks of remote IPs. An undergraduate perspective, Meredith Theus, Reed ‘21**

The ability to ask and answer questions through experimentation and research is central to the study and application of ecology; thus, IPs provide students with the opportunity to fully understand and internalize the materials and topics covered in an ecology course. IPs act as a total synthesis of the course materials relying on adaptation and problem‐solving that an examination cannot provide. Due to the scope and novelty of IPs for many students, it is important to emphasize student–student and student–professor communication while preserving the “independent” aspect of IPs. Communication helps determine the scope and recognize the practicality of completing a project, demonstrates to students how a professional in the field asks and answers questions, and models a real‐world experiment where communication is a necessity. However, the independent in independent project is crucial. The ability to question and investigate for one's self is important in developing experience, interest, and curiosity in the outcome of the research. As time management can be harder during remote learning, interest in the research as well as a plan and reasonable scope determined through communication are central to completing the project. A lack of equipment and materials also poses a challenge to completing IPs remotely; however, the ability to conduct field work and use pre‐existing data and databases that ecology allows for is a way to minimize this challenge. Ultimately, IPs provide an opportunity for students to learn, synthesize, and problem solve in a way that prepares for and models professional research in the field of ecology.

### 2.4 Project dissemination modifications

The final week of projects was focused on dissemination and communication of project findings, a critical component of inquiry‐based learning (Schamel & Ayres, 1992; Symes et al., 2015). Bio 301 culminated in an IP symposium, where each group presented a formal 6 min presentation followed by 3 min of questions from their peers. Presentations and the following Q and A were completed over Zoom using screen sharing options (Box 2). Additionally, students were required to submit a project abstract in the format required for submissions to the Ecological Society of America's 2020 meeting, also requiring one key display item and associated caption and any data, metadata, and code used in the analysis of the project. The quality of presentations, including the feedback given by students during digital presentations, was comparable with previous offerings. While all student participants had access to reliable high‐speed Internet in this course, future consideration should be given to making alternative formats available to accommodate students who are unable to access reliable Internet. Additionally, all students participating in this project were able to participate synchronously across laboratory weeks; however, future designs should incorporate the possibility of asynchronous remote IPs.

Box 2 **Useful tools and resources for completing remote independent projects**

**Virtual Meeting Software (e.g., Zoom, Google Meet, Skype)**

The COVID‐19 pandemic prompted a crash course in virtual meeting software usage among academics in order to maintain lines of communication with our students, colleagues, family and friends. It has become an essential tool for conducting much of our work but is especially important for virtual classroom interactions between faculty and students and also student to student. In addition to the ability to see and interact with students “face‐to‐face,” many of the virtual meeting software platforms have key features that are particularly useful for completing independent student research projects. For example, the ability to share screens during live video conferences provides an easy way for students to give presentations to their peers and faculty (Table 1). Additionally, features that allow instructors to place students into virtual “breakout rooms” can facilitate small group discussions within project teams which can be an indispensable tool that allows instructors to listen in on project discussion and provide feedback to project groups, and even call students back into the main room for larger group discussions or sharing of general information/feedback. Finally, the built‐in ability to record class discussions and virtual project work can allow for asynchronous participation of some class members.

**VPN access to campus‐based resources**

One of the key challenges of the COVID‐19‐driven shift to remote learning is the loss of access to on‐campus resources (see below). This is especially important for specialized research software programs and other computational resources that may be impossible or cost prohibitive to run on individual student's computers. Virtual Private Network (VPN) connections can provide an important means for students to connect to on‐campus computing resources and software. For example, students in the ES 300 course were able to connect to computers running ArcGIS software (Ersi, Redlands, CA) in order to complete independent research projects requiring GIS tools. VPN connections can also be leveraged to connect students in remote working environments to campus library resources for literature review work as part of independent projects.

**Cloud‐based file management, document editing and coding**

In a remote learning setting, challenges abound for finding synchronous periods of time for student collaboration. Cloud‐based services that allow for shared file management and document editing (e.g., Google doc, Google slides, Google sheets, GitHub) are an essential tool for team‐based independent projects. These tools are freely available to all students and can facilitate all aspects of the project process, from planning to dissemination. In the remote work environment especially, these tools provide distinct advantages over locally stored file systems. When in person, students working groups can work from a single computer to contribute to a particular project‐related assignment but this is not possible in remote learning situations. The cloud‐based options mentioned here can allow for students in different locations to easily collaborate by simultaneously viewing and editing an assignment from different machines and locations. This is essential for the completion of group assignments in independent projects.

**Learning management systems**

Most institutions of higher education have site licenses for proprietary learning management systems (e.g., Moodle, Blackboard, Google Classroom) that many faculty use to share resources with students, create, collect, and grade assignments, and many other course related tasks. These systems can be particularly useful in remote working environments. This is especially true if students are already integrated into these systems and access course content through these systems. In the context of remote IPs, these systems can be useful for sharing information about the IPs, collect project‐related assignments, and provide a one‐stop‐shop for accessing other tools and resources needed for completion of the IPs.

**Delivery services**

Many institutions will have their own mailing and delivery services which provide avenues for transporting scientific equipment between campus and student residences. In our experience, purchase of relatively low cost ecology equipment (e.g. calipers, field tape measures, flagging tape) directly from supplies can be more cost effective than mailing items from campus. Importantly, a plan for student responsible during the activity, and eventual relocation to campus should be established on the onset of IPs.

### 2.5 Student evaluations

**FIGURE 1:**
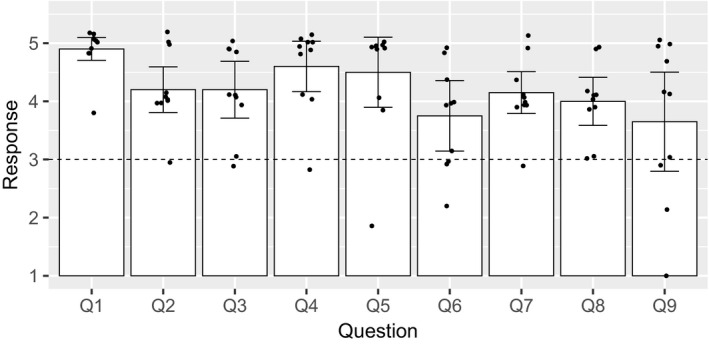
Student evaluations of queries related to independent projects. The student response (mean ± 95% confidence intervals) to nine questions regarding the effectiveness of the independent projects (*N* = 10 students). Point represents the responses of each student to each question. Responses indicate 1 = “strongly disagree,” 2 = “disagree,” 3 = “neutral,” 4 = “agree,” 5 = “strongly agree”, where the dotted horizontal line indicates a neutral response. See Table 2 for specific queries

Following completion of Ecology IPs, students were anonymously surveyed on their perceptions of the effectiveness of this activity using the survey administration application Google Forms (Google LLC). Of the students who completed an IP, we obtained 10 responses (100% completion) for all students’ queries, and all students responded to all quantitative questions. All responses were also obtained within the same day that the survey was administered. Students responded to nine queries (Table 2) with a numeric response to each question ranging from “strongly disagree” (1) to “strongly agree” (5) (Figure 1). Indeed, self‐reported data falls short of the gold standard for measuring impacts (Critcher & Dunning, 2009), and future iterations where schedules are less unexpected should rigorously explore any efficacy of novel teaching techniques. How such outcomes compare to other potential uses of class and laboratory time have not been empirically explored but are important alternative models (e.g., structured online laboratories) that should be used to determine whether engaging in such an activity optimally targets learning outcomes amidst distancing. Additionally, a lack of comparable data of previous “on‐campus” iterations precludes an understanding of how much learning varies relative to traditional years and remains a research opportunity for future years.

**TABLE 2:** Queries (Q1‐Q9) posed to students to evaluate perceptions of the Bio 301 IPs, as well as the student mean response (*N* = 10 students), 95% confidence intervals in brackets

Query	Statement	Mean [95% CI]
Q1	I felt safe while completing my ecology independent project.	4.90 [4.7, 5.1]
Q2	This activity increased my knowledge of local natural history.	4.20 [3.81, 4.59]
Q3	This activity increased my familiarity, and ability to engage with, the primary scientific literature.	4.20 [3.71, 4.69]
Q4	This activity gave me an appreciation for the challenges of connecting a hypothesis with a plan to test my hypothesis.	4.60 [4.17, 5.03]
Q5	This activity increased my familiarity with how to design ecological experiments.	4.50 [3.90, 5.10]
Q6	This activity allowed me to increase my statistical and/ or computational proficiency.	3.75 [3.14, 4.36]
Q7	This activity increased my understanding of local ecological processes.	4.15 [3.79, 4.51]
Q8	This activity increases my ability to communicate scientific results during an oral presentation.	4.00 [3.59, 4.41]
Q9	I felt that being off‐campus reduced the quality of my independent project.	3.65 [2.80, 4.5]

Note

Responses indicate 1 = “strongly disagree,” 2 = “disagree,” 3 = “neutral,” 4 = “agree,” 5 = “strongly agree.”

The survey results provide useful initial feedback. Importantly, student perceptions of their safety while engaging in IPs suggest that such projects can be conducted safely (Table 2, Q1, mean = 4.9). A lower outcome here would make us unwilling to repeat this activity, irrespective of the other outcomes. Continued efforts toward ensuring student safety depend on a consistent updating of facts on the ground and dialogue with students. Importantly, and paradoxically, proximity to other humans presents both potential danger (e.g., increasing the chance of acquiring infectious diseases) and a necessary ingredient to ensure safety (buddy system).

Responses to queries 2–9 indicate that students perceived this activity to increase some benchmarks of scientific competency, as envisioned on the onset of this experience and assessed relative to a neutral response (response = 3). For examples, students generally agreed with the activity's ability to increase: knowledge of local natural history (Q2, mean 4.2) and local ecological processes (Q7, mean 4.15), familiarity with the primary literature (Q3, mean 4.2), experimental design (Q5, mean 4.5), and scientific communication skills (Q8, mean 4.0). One additional goal of this activity was to give students an appreciation for the difficulties of connecting a hypothesis with a plan to test the hypothesis (Q4, mean 4.6). Indeed, many of the ecological studies routinely presented in lecture to reinforce ecological concepts (i.e., textbook examples) suggest once a hypothesis is formulated, it is trivial to design and implement a plan to test it (Windschitl et al., 2008).

Students additionally expressed a wide range of perceptions on the extent to which being off‐campus reduced the quality of their independent projects (Q9; Figure 1). While the confidence intervals for the mean response overlaps with a neutral score of 3, the majority of students (60%) reported that being off‐campus reduced the quality of their independent project to some extent (Q10, mean 3.65). Finally, the goal of “increasing computational and statistical literacy” scored lower relative to the other queries (Q6, mean 3.75). Previously in class, we had described this term narrowly to describe familiarity with the use of the R statistical environment and the use and knowledge of statistical tests. This low Q10 score may be due to a limited collection of data or that data collection occurred late in the IP process, precluding substantial amounts of time to be spent on statistical and graphic analyses, or may be a result of students underestimating their own learning in this area due to underestimating the value of more basic computation tools in research.

## 3 CASE STUDY 2: ENVIRONMENTAL STUDIES JUNIOR SEMINAR (ES 300)

### 3.1 Overview

The Junior Seminar in Environmental Studies (ES) at Reed, ES300, is an interdisciplinary, problem‐oriented course that all ES students take in the second semester of their junior year and is co‐taught by faculty from the natural and social sciences. The ES major at Reed is designed for students that seek a broad training in environmental themes that is anchored by a strong disciplinary focus in one of 5 major departments: biology, chemistry, economics, political science, or history. Each year in ES 300, students from these disciplines come together to form an interdisciplinary team that will work together during the semester with the course's faculty and a local stakeholder to design and implement a student‐led research project that aims to inform the climate change‐related work of the stakeholder. During the Spring 2020 semester, the course consisted of 20 students from biology (*N* = 5), chemistry (*N* = 3), political science (*N* = 5), economics (*N* = 3), and history (*N* = 4). The ES 300 students worked together to conduct a climate change vulnerability assessment of Portland's natural areas for the City of Portland's Bureau of Environmental Services. The project was designed to allow students to build skills in Geographic Information Systems (GIS), quantitative data analysis and interpretation, primary literature review and synthesis, as well as working as part of an interdisciplinary, collaborative team to address an environmental problem. During the coronavirus pandemic‐induced move to online learning in the spring of 2020, the ES 300 students all relocated to off‐campus working environments and were asked to complete this project and deliver it to the stakeholder. The following documents the changes to the planning, implementation, and dissemination phases of their project and highlights key themes from the course's student evaluations of this experience.

### 3.2 Project planning modifications

Prior to the move to remote instruction, students were presented with a “problem” and research challenge from the local stakeholder that would require an interdisciplinary research approach to solve. The students were then asked to work together to propose an approach to solving the problem that integrated biophysical, ecological, political, economic, and historical perspectives. This large group project is designed to create a *cooperative learning environment* and *positive interdependence* (Laal, 2013) within the class as students work together to achieve this mutual goal. Just as this proposal process was getting underway, the school moved to an all online mode of instruction for the remainder of the semester. A major concern of faculty at this point was how to cultivate the kinds of peer‐to‐peer interaction that are crucial to cooperative learning and positive interdependence in a remote learning environment. Faculty decided to address this by breaking the students up into smaller interdependent teams of 4–5 students and employ a “jigsaw” cooperative learning approach (Colosi & Zales, 1998) that would allow each team to specialize on one aspect or “chapter” of the project proposal that would then be integrated with the work of other groups to produce a single project proposal. Each group was also asked to give an informal proposal presentation to the class during a synchronous class meeting via Zoom using the program's screen sharing features.

In an effort to assure small groups of students were as functional and interdependent as possible, we used the survey program CATME Team‐maker (Layton et al., 2010) to select student teams that had similar schedules and working habits but diverse disciplinary backgrounds. Finally, we devoted initial synchronous class meetings via video conference (Zoom) to discuss the importance and strategies for working in interdisciplinary teams and pointed out the similarities between our remote working environments and those that many career environmental scientists use every day. The goal of fostering this conversation was to gain “buy in” from the students and further build positive interdependence.

Finally, it should be noted that students were encouraged to use existing datasets that were readily available via public data repositories. While primary data collection and analysis is often a learning objective of this course, the realities of the coronavirus‐related lockdowns precluded this option for our students. Additional focus was placed on how to find, access, and use existing, publicly available datasets.

### 3.3 Project implementation modifications

The project proposal students submitted required them to use extensive GIS analysis. To accomplish this in a remote working environment, students were guided through a process of setting up Virtual Private Network (VPN) access to on‐campus computing resources and library resources needed to complete their work, requiring contact and support from campus computer and library services. We also set up a Slack (Slack Technologies) workspace for students to encourage asynchronous collaboration and communication throughout the project, with channels to organize the conversations of small groups as well as the larger group conversations about the full report.

For many student groups, scheduled synchronous class and laboratory periods were rare occasions for which all group members were available. Therefore, we dedicated scheduled class meetings to allow for students to meet and work on project. This was facilitated using the “breakout rooms” feature on Zoom (see Box 2), which allowed faculty to both provide a working environment for each student group and address the whole class as needed for general information/feedback. This meeting format also allowed for faculty to consult with project groups and provide feedback directly by “walking” from virtual room to virtual room. Irregular check‐ins outside of class time and office hours were additionally made available as well to help maximize mentoring opportunities during this phase of the project. These more frequent meetings proved helpful to field questions that arose during data collection and analysis.

Students were additionally asked to provide weekly, informal “progress reports” to the larger group. These reports were given verbally during scheduled class times and, like the informal proposal presentations, provided opportunity for peer‐peer evaluation of the projects and feedback, as well as group problem‐solving of any issues that arose during the implementation of the project plans.

### 3.4 Project dissemination modifications

In the face‐to‐face version of ES 300, students make a formal presentation of their project findings and deliver a hardcopy of the project report to the stakeholder during a symposium that serves as the final class activity. The online version of the course this spring followed a similar structure, but the presentations and report were delivered electronically (via Zoom and email, respectively) to the local stakeholder. The communication of research findings outside of the academic community is a key learning objective for this interdisciplinary CURE (Jones, 2009).

### 3.5 Student evaluations

The ES 300 class was given a survey at the end of the remote group project assignment via Google forms. Questions were open‐ended and encouraged students to provide (<300 word) responses that evaluated their own roles and performance, their peers, and their overall experience with this assignment (See Appendix 3). All 20 students completed the survey. Here, we provide a summary of the responses to the question(s) about their overall experience, organized around several recurring themes and supported by illustrative quotes.

### 3.6 Theme 1—Experience working in remote teams is relevant to career aspirations

Most students mentioned the utility of the remote working environment in providing experience that will be relevant in their future academic and professional careers. We discussed this goal as the class transitioned to a remote working environment, and these responses suggest the project was an effective means to achieving these ends. Given this response, we recommend highlighting the connection between the remote classroom environment and the likely future working environments students will encounter. Illustrative quotes:

- “this project was good experience into how most of remote, collaborative ES work is done in the ‘real world’, and what it is like working for a stakeholder.”
- “This project was the first big experience I had with remote group projects, so I think that was really useful and will definitely come in handy after Reed.”
- “I think this class was one of the most helpful of all my time at Reed at showing me real world and hands‐on applications of my major.”

### 3.7 Theme 2—Students can gain identifiable, valuable skills in conducting remote, collaborative work

Many students were able to articulate areas of skill growth as a result of the remote, team science experience in the virtual classroom. In particular, skills related to communication, setting of goals/expectations as a group, and navigating challenging group dynamics were cited. Illustrative quotes:

- “I found the practice in remote collaboration to be newly difficult but curiously conducive to different strategies for delegating and dividing the workload, given the unique challenges and unexpected perks of working from afar.”
- “I think I have a much better idea of what it takes to accomplish something important and I have much more confidence in myself that I can collaborate [with] people in the future”
- “I feel more comfortable working on group projects and am more able to navigate difficult group dynamics.”

### 3.8 Theme 3—The challenges presented by the remote learning environment were navigable with help from more experienced peers, faculty, and technology

Most students acknowledged some difficulty and challenges with the remote working environment but were also able to articulate how those challenges were largely overcome. In many cases, the fact that students were working in interdisciplinary teams allowed for them to lean on the skills of more experienced group members for particular tasks and find ways to solve problems together that would have been difficult or impossible to do on their own. This is consistent with creating a cooperative learning environment and “zone of proximal development” (Amalia, 2018) and suggests that placing students into these complimentary, interdependent groups can help with overcoming some of the challenges students face with solo remote learning. Illustrative quotes:

- “Having to work remotely was certainly a challenge but I think that we were able to manage this as a group and found ways to collaborate and communicate.”
- “I became closer with people in the class which I hadn't known as well before. I really appreciate group projects and think collaboration is a huge part of science.”
- “The online situation really added some difficulties in terms of making sure everyone was on track and had a good idea of their part of the project. I think we made good use of email, Slack, and Zoom though…”

### 3.9 Theme 4—Not all students were able to navigate the remote working environment successfully

While the majority of our students reported finding success in overcoming the challenges of the remote working environment (Theme 3), not all did. These challenges were not always apparent until this final evaluation. Therefore, we recommend early efforts to identify and support these students with additional faculty involvement.

Illustrative quotes:

- “I learned that doing group projects over zoom is very difficult. I will definitely be more grateful to have classroom group time in the future.”
- “It is really hard to remain in the loop when we are only talking over video chat every now and then.”

## 4 SYNTHESIS AND CONCLUSIONS

We identify major challenges as well as opportunities associated with implementing remote CUREs based on our experiences in Spring 2020. First, safety is paramount, is not to be taken lightly, and can be harder to ensure remotely. Secondly, physical materials can help facilitate projects, but require early action that might constrain the independence of student projects. Delays involving shipping items from campus to students, and completing online purchases present serious impediments to project implementation (Box 2). Potential solutions involve reliance of digital tools and existing data, constraining the scope of projects and the set of materials required, limiting project planning to a single week to increase time dedicated to implementation, or extending the total duration of the IPs. Third, given the novel academic setting, projects may be reduced in scope (e.g., yield less data) from what might typically be feasible on campus in the same time. Here, we recommend calibrating expectations by emphasizing the importance of process and skill building rather than outcomes: combatting the perennial challenge in science education that obtaining “clean” data (e.g., low variance surrounding treatment means) or data that support hypotheses should be the primary outcomes by which the merits of the project are assessed (Symes et al., 2015). Finally, in the absence of regularly scheduled periods where students and faculty work in close proximity in a field or laboratory setting, creating meaningful student‐faculty interactions must be re‐imagined, yet prioritized.

While the experience of conducting CUREs in remote learning environments is challenging, there are also several distinct opportunities we observed for advancing learning objectives. First, the execution of original research in distributed working teams adds an authentic 21st century experience for future ecologists and environmental scientists. Many modern ecological or environmental science projects are carried out by scientists collaborating across institutions, regions, countries, and disciplines (Hampton & Parker, 2011). Therefore, the need to navigate the communications, tools for collaborative data collection/analysis, and preparation of research products provides real‐world experience that students will need when they enter the scientific/academic workforce. These are precisely the kinds of “learning by doing” experiences valued in modern academic institutions (Metzger & Langley, 2020).

Additionally, the remote environment creates unique opportunities for students to engage in science outside the walls of the “Ivory Tower” and with particular constraints that may serve to enrich the experience. For example, a student may develop an important and novel “sense of place” by completing a research project in their hometown, away from their academic institution while working from home. Working from this “nonacademic” environment presents constraints on research, such as access to simple, nonspecialized equipment, but such constraints may encourage creative, accessible research approaches (Acar et al., 2019) that would be missed in more traditional academic settings. Furthermore, the remote situation presents additional opportunity for students to engage with a larger pool of nonacademic partners or “stakeholders” (e.g., ES 300 class) that might be unable to devote the time to meet and interact with students’ in‐person but can afford the time to engage in one or more virtual meetings with students.

A final silver lining of remote CUREs involves mentoring, a critical component to maximizing outcomes during CUREs (Linn et al., 2015), which can be achieved with modern tools such as virtual meeting software and learning management systems (Box 2). The ability to schedule virtual “face‐to‐face” meetings between whole classes, small groups and individual students and faculty provides a more than adequate setting for the exchange of ideas, sharing of experiences, and relationship building that are critical elements of mentoring (Symes et al., 2015). Likewise, other essential aims of CUREs, such as moving outside of textbook examples and participating in the scientific method (Windschitl et al., 2008), still remain possible in a remote setting.

Instilling students with an understanding of what doing science entails remains among the most important aims pursued by science educators. While continuing this quest in the COVID‐19 era requires flexibility and a reassessment of what comprises essential curricular elements, we remain optimistic that institutions of higher education can remain committed to the process of teaching students how to develop new knowledge, work collaboratively, and build critical skills through active engagement in the scientific process. Importantly, there are many research questions yet to be answered regarding the efficacy of remote CUREs that require future study and are beyond the scope of this effort. Yet, it is our hope that our experience, as written, provides educators with a framework for conducting remote CUREs that contribute to this mission.

## CONFLICT OF INTEREST

The authors have no competing interests to declare.

## AUTHOR CONTRIBUTION

**Samuel B. Fey:** Conceptualization (equal); Data curation (equal); Investigation (equal); Project administration (equal); Writing‐original draft (equal); Writing‐review & editing (equal). **Meredith E. Theus:** Investigation (equal); Project administration (equal); Writing‐original draft (equal); Writing‐review & editing (equal). **Aaron R. Ramirez:** Conceptualization (equal); Data curation (equal); Investigation (equal); Project administration (equal); Writing‐original draft (equal); Writing‐review & editing (equal).

## Data Availability

The data associated with this manuscript are included within the manuscript.

## Appendix 1 Bio 301 Spring 2020 IP assignment materials, conducted at Reed College in Spring 2020

### Independent projects synthesis assignment (Due via Email by 0700 Pacific Time on Thursday, 30 April)

#### Preface to S20 independent projects

As we've seen throughout the semester, ecologists are fortunate in that it is feasible to produce high quality science without a large budget. For example, Colin Donihue's recent *Nature* paper we discussed on hurricane‐induced selection on *Anolis scriptus* was accomplished through the use of an iPhone 7 and a leaf blower. This type of study exemplifies that the limiting factor for good science is often identifying an important, but understudied, topic. We've also seen that ecology can be conducted in environments with both low and high amounts of human impact. As such, while the members of our class will be geographically spread‐out for the final five weeks of the spring semester, and in many cases lack access to much formal scientific equipment, I am confident that we will be able to actively engage with the process of “doing” ecology, and ask and answer some interesting ecological questions.

On the onset of this assignment, I want to stress your safety in this endeavor. Under no circumstances, do I want this project to put you into any dangerous situations. This could mean having you come into crowded areas containing people, but this could also mean working by yourself in a remote setting. In all cases, be sure to discuss and have plans greenlighted by me in advance of any work. Along these lines, I will also reiterate that there are three legs to the stool of ecology (sensu Cottingham et al., 2017): observations, experiments, and models. Observations can be made firsthand, but existing observational data (which abundantly exists online) can be leveraged to answer question. The option to use an existing model, or build your own model, to answer an ecological question is often an overlooked approach that requires no travel and no equipment.

You can complete this assignment individually or in groups. I strongly encourage to work in groups. One nice feature about the available tools for online collaboration (Zoom, Skype, Slack, Google Drive, Drop Box, etc.) is that it is possible to collaborate with other group member even if you are geographically separated. In fact, it could actually be a benefit to have group members in different locations if you are asking a question that pertains to certain factors (e.g., latitude, photoperiod, temperature)! How big can/should a group be? There are no maximum sizes here, but the number of team members should scale with the complexity of the problem.

A few thoughts on what can work well for a project. (1) Start with a concept or theory that we've covered in class, then think about what study system you might be able to use to investigate this theory (e.g., optimal foraging theory makes specific predictions about individual and group behavior that could be investigated by having access to a park area where ducks or squirrels feed). (2) Start with a project that we've investigated during the first 7 weeks of laboratory and think about how you might expand on it (mimicry extensions could easily be accomplished with household equipment). (3) While we've halted our phenology project that focused on covering a range of focal trees across a long amount of time, it's certainly possible to ask a phenology question that substitutes space for time and many interesting life history stages will be unfolding in the next two months.

As we move forward, I’ve created a starting timeline, which we can modify as need be:

*Week 1 (1‐Apr, 2‐Apr)*: In‐lab project brainstorm and idea sharing. To help get off the ground, come prepared to spend ~5 min outlining a potential IP idea. During your project pitch, focus on (1) what is the motivation for doing this project (does it address a meaningful applied question? Does it address an understudied problem fundamental for understanding ecological processes?), (2) what the data/data product collection would look like, (3) what are your hypothesis (or predictions, expectations)? and (4) how might you statistically analyze your data. After each project presentation, we will hold a Q and A from the rest of laboratory to further clarify the projects. At the end of this brainstorm, we will have time to allow group fusion, and I would be excited if some of the presentations are exciting enough to have multiple people pursue the same focal question.

*Week 2 (8‐Apr, 9‐Apr):* Project check in. Each group will give an update of what they are planning to pursue for their IP, and give a more detailed version of the focus of week 9. If any pilot data have been collected, these data can be shared at this time. After each update, other groups and myself will give feedback on how projects might be improved, or ask clarifying questions. At the end of this time, all individuals should be in a group, materials needed to complete the project and have a viable project designs and timeline to complete their projects.

*Week 3 (15‐Apr, 16‐Apr)*: Project work time. Project check‐ins can be scheduled anytime during the scheduled laboratory meeting, other days as needed.

*Week 4 (22, 23‐Apr)*: Project work time. Project check‐ins can be scheduled anytime during the scheduled laboratory meeting, other days as needed.

*Week 5*: Project presented in class on 30‐Apr (see below).

### Project components (3 parts described below)

#### Part I: Scientific abstract

Scientific abstracts epitomize the need for scientists to communicate their findings clearly and succinctly. While most abstracts are limited to a few hundred words, their importance is difficult to overstate. When appearing at the start of a manuscript, a well‐written abstract will help explain to journal editors why your manuscript is worth publishing, and once published will entice readers to invest time into reading the rest of your paper. Additionally, most scientific conferences require the submission of abstracts that determine whether you are allowed to present your research. As part of your final projects assignment, you will be writing a scientific abstract for submission to this year's Ecological Society of America (ESA) Meeting based on the results you obtained during your independent projects. Below are the criteria for this assignment (adopted from the ESA website). For examples of one such abstracts, please read this year's ESA meeting website: https://www.esa.org/saltlake/program/abstracts/call‐for‐contributed‐oral‐abstracts/

- Abstracts may be no longer than 400 words in total and must include information on
  - *Background/Questions/Methods*, using up to 200 words to identify the context, objective and any salient methodologies of the study.
  - *Results/Conclusions*, using up to 200 words to explicitly report the results, interpretations and implications of the study.
  - *One key figure* (may be a multi‐panel figure) *or table* capturing the take home message of your study, including an informative caption. Abstracts without explicitly stated results will be rejected.
- Please include the use of inferential statistics in your *Results/Conclusions* section.
- Citations are typically not included in scientific abstracts, and should not be included for these submissions.
- Be sure to include a project title and list of authors. When entering your title, capitalize the first word, proper nouns, and the first word following a colon. The title is limited to 255 characters (about 15 words). Do not type the title in all capital letters. Place a comma before the word “and” in a series. Do not end your title with punctuation
- For each author, enter the full first name, the first letter of the middle name, and the full last name. Do not add punctuation after any of the names.
- Producing a well‐written abstract is difficult and time consuming. Please start early, ask Sam questions, solicit feedback from other groups, etc.

#### Part II: Conference presentation

Many scientific conferences are comprised of scientists communicating research findings using short digital presentations followed by a brief question and answer period from the audience. For the final class period (Thursday April 30), we will be hosting a research symposium that will feature presentations on your independent projects. The format will be as follows: 6‐min research presentation followed by 2–3 min of audience questions for each group. This is not very much time to present, so please carefully think through how best to use your presentation time (I’ll also be strict about cutting off presenters at 6 min). The format for these presentations is completely open ended. The most effective presentations will provide the necessary background information that motivated the research effort, a clear statement of the question investigated, a brief methods overview, the key results, and perhaps most importantly, a discussion of what these results mean. Please come speak with me early if you have questions about how best to use your time. Additionally, for audience members, the Q and A time is a great chance to hone your ability to ask thought provoking questions. Similar to the abstract, please send via email, your slides no later than 0700 on April 30.

#### Part III: Data/ metadata

As we've discussed this semester, one of the hallmarks of science is that it is reproducible. Along these lines, please submit your data, metadata, and any code that you've collected during this project. How you submit these files is up to you.

## Appendix 2 Additional project details for Bio 301 IPs

**TABLE A1:** Student project titles, the number of students working on a project, the geographic location(s) of student(s) (all USA), and the additional materials required to complete project

Project Title	*N* students	State(s)	Materials needed
Avian foraging and the warning signal hypothesis	2	IL, ID	For each location: 2 digital cameras, rechargeable batteries, window screens, bitter spray
An exploratory study into foraging strategies and food resource competition in Pacific Northwest squirrels	1	WA	Calipers, field tape measure
Modern impediments for *Camassia quamash* in the west	1	OR	Digital Camera
Creek alteration and macroinvertebrate characteristics	1	WA	Invertebrate D‐net, dissecting microscope and light source, 20‐ml glass containers, gridded counting dish, squirt bottles
The study of cochineal density patterns on a Southwestern Cactus Species, *Opuntia ficus indica*	1	CA	NA
Foraging Behavior of Steller's Jays in Predator‐Prey Interactions with Sharp‐Shinned Hawks	1	CA	2 trail cameras, mesh for feeding arrays
Mimicry 2.0: How good do I need to look?	1	WA	Window screens, bitter spray
Effects of color, and human presence on the feeding decisions of birds	1	CA	NA
Directionality and bat nesting preferences in the American mid‐Atlantic	1	VA	NA

## Appendix 3 ES 300 Spring 2020 IP assignment materials

### Syllabus Sample: ES300—Spring 2020

**Junior Seminar in Environmental Studies**

Brian Tyrrell and Aaron Ramirez

Reed College, Portland, OR

### Course description

ES300 is an interdisciplinary, problem‐oriented, and project‐based class. The first part of the course is dedicated to building a shared, interdisciplinary knowledge base on the topic of climate change. In particular, we will study the role environmental science and other forms of academic work play in shaping policy, informing resource management, and influencing public perception of climate change. In the second half of the course, students will work together with a local stakeholder to design and implement an independent research project that aims to inform the climate change‐related work of the stakeholders.

### ES300 Group Project (Spring 2020)

Natural Areas in urban environments are a critical component of urban infrastructure that provides many natural benefits to urban populations. Understanding and harnessing these benefits has never been more important given the realities of a changing global climate, increasing human populations, and the expanding footprint of cities worldwide. The goal of this year's ES300 group project is to develop and address interdisciplinary research questions related to the impacts of climate change on Portland's natural areas and the human/nonhuman communities they support. To accomplish this, students will work with Portland's Bureau of Environmental Services to generate, collect, and analyze data on Portland's urban natural areas undergoing active revegetation projects. The project will allow students to build skills in Geographic Information Systems (GIS), quantitative data analysis and interpretation, primary literature review and synthesis, as well as working as part of an interdisciplinary, collaborative team.

- *Schedule*

**Table:**

Activity	Date
Proposal discussion	Tuesday March 31, 2020
Proposal due	Wednesday April 1, 2020
Proposal presentations	Thursday April 2, 2020
Group work	Tuesday April 7, 2020
Group progress reports	Thursday April 9, 2020
Group work	Tuesday April 14, 2020
Group Progress Reports	Thursday April 16, 2020
Ch. reports due, full report disc.	Tuesday April 21, 2020
Full report work/formatting	Thursday April 23, 2020
Full report due, practice delivery	Tuesday April 28, 2020
Project delivery to BES	Thursday April 30, 2020
Self & Peer evaluation	Wednesday May 6th, 2020

- *Proposals & Proposal Presentations* ***—*** As discussed before the break, the proposal needs to include the following components: Background, Research Question/Problem, Methods/Approach, List of needed data/documents, and lit cited. To help in formatting and collecting this info, we've created this Google form. Feel free to enter your information into the form or use the “file upload” link at the bottom of the form to submit your preformatted proposal. These proposals are due Wednesday April 1st by 5 p.m. (PDT). This is not meant to be a daunting assignment. It will mainly serve to provide us with a window into the plans of your group and help us with planning to support your group projects. In class on Thursday, you will present your proposed project to the class via Zoom. Each group will have 8 min to share your project idea and proposed work plan. This will be an update on the ideas you shared before break and help with moving forward on your projects. We will open things up for questions/discussion from the class. We recommend putting a couple slides/visuals together to help with communicating your ideas to your peers using the “screen share” feature on Zoom but this is not required.
- *Group work*—We will use our scheduled in‐class time to work within your groups to advance the objectives of the class project. We will facilitate this using Zoom's Breakout Rooms feature, which we tested out before the break. Each day that includes “group work” will begin with a large group Zoom call and subsequently break off into project groups. The breakout groups will be called together for larger group discussions as needed. One advantage of using in‐class time for group work is that Brian and Aaron will be readily available to field questions and work with you to advance your project during this time. If helpful, we can create goals with you and your group for how to use this time most effectively.
- *Group Progress Reports*—Twice during the project period, groups will provide a “progress report” to the full class. This will be facilitated using a Zoom call that includes all members of the class. Each group will take turns providing the class with an update. You are welcome to use screen sharing to show any maps, data analyses, summary documents, etc. This will provide some accountability for making progress on your project week‐to‐week as we move toward the final deadline and will also provide opportunities for peer and instructor feedback.
- ***Chapter Reports*** —As mentioned in class, we are producing a single report but with 4 distinct “chapters” based on the group‐level work you all are doing. These chapter reports will each include an Abstract, Introduction/Background, Methods, Discussion, Lit Cited, and Summary of any management recommendations. More information on the chapter report format will be discussed in class. These chapter reports will need to be completed prior to final project delivery to allow for incorporation into the full report.
- *Full report*—The ultimate product of the project is the full report that will be delivered to BES. This will be composed of the chapter reports + a title page, Executive Summary, Acknowledgements, and any Appendices (additional maps/figures, methods descriptions, datasets, etc.). The full report will be due on the second to last class meeting, in time to review before the electronic delivery to BES.
- *Project delivery*—Typically, we invite the stakeholder to campus and physically “deliver” the report and provide a PowerPoint‐style presentation of the major findings. This year we will conduct our project delivery in a virtual format via a Zoom Webinar. You will give a presentation of your report to our stakeholders at BES on the date outlined above. In addition, we will electronically deliver the report to them. This will constitute the “final examination” for this course.
- *Self and Peer evaluation*—As outlined in the syllabus, you will complete a self and peer assessment of your performance during the project phase of the course. This is a key opportunity to reflect on this experience and offer insight that will aid you in evaluating what you've learned, as well as helping us to evaluate this aspect of the course. This is a mandatory assignment in order to successfully complete this course.

#### Group Communication is key

With the reality that we will all be working remotely for the remainder of the semester our means of communication will all be electronic. Many tools exist for facilitating remote working groups. The tools we recommend are:

- *Google Drive* ***—***strive to work on shared documents through the college's Google tools as much as possible. We have unlimited file storage capacity and these services are well integrated into Reed's computing ecosystem. You can share docs, spreadsheets, presentations, GIS files, maps, papers, etc.
- *Zoom/Google Hangouts*—You are welcome to organize virtual group meetings through Zoom (Reed documentation) and Google Meet (Reed documentation) for free. The free version of zoom only allows 40min meetings but they can just be restarted if you run into that limit. Google Meet is unlimited.
- *Slack*—We have created a Slack workspace to help facilitate electronic communication within and across groups. Create your own group Slack #Channel to facilitate discussion within your group and use the shared channels to communicate across groups or with your instructors (see below).

#### Sample questions from Self/peer evaluations

1. Please describe your role in the group project. What was your primary contribution(s)? Where did you collaborate with other students? Where did you take a leadership role? What work did you do that may not have ended up in the report itself? [Limit to 300 words]
2. Please EVALUATE your contribution to the group project. What did you do well, and what could you have done better to facilitate the group's success? [Limit to 300 words]
3. What did you learn/gain from the experience working on the group project this semester? How do you think it will help you moving forward at Reed and beyond? [Limit to 300 words]
